# Developments in Polymer Theory and Simulation

**DOI:** 10.3390/polym12010030

**Published:** 2019-12-23

**Authors:** Martin Kröger

**Affiliations:** Polymer Physics, Department of Materials, ETH Zurich, Leopold-Ruzicka-Weg 4, CH-8093 Zurich, Switzerland; mk@mat.ethz.ch

Polymer theory and simulation are topics that are still growing (Figure 1), highlighting the need for an understanding of microscopic mechanisms influencing material properties of systems containing macromolecules. While there is a clear tendency for a growing industrial interest in biodegradable polymers and speciality polymers, many questions related to the behavior of polymeric systems can be tackled using theoretical approaches that apply to most kinds of polymers. Polymeric materials display distinguished characteristics which stem from the interplay of phenomena at various lengths and time scales. Further development of polymer systems critically relies on a comprehensive understanding of the fundamentals of their hierarchical structure and behaviors. Composite materials containing polymers offer a huge diversity that is currently explored. With the help of increasing computational resources, the study of entangled polymers is, nowadays, within reach. Multiscale approaches are developed and investigated in parallel. This editorial aims at highlighting some of the developments reflected by articles published in *Polymers* since 2017.

Multiscale Approaches

As discussed in detail by Gooneie et al. [1], there are three main categories of multiscale approaches: (i) sequential, (ii) concurrent, and (iii) adaptive resolution schemes. The (i) sequential approach (also known as the implicit, serial, or message-passing method) links a series of computational schemes in which the operative methods at a larger scale utilize the coarse-grained (CG) representations based on detailed information attained from smaller scale methods. The bridging of various scales in a sequential method is often implicit. (ii) Concurrent (also known as parallel or explicit) methods are designed to bridge the suitable schemes of each individual scale in a combined model. Such a model accounts for the different scales involved in a physical problem concurrently and incorporates some sort of a handshaking procedure to communicate between the scales. (iii) Within an adaptive resolution scheme approach, single atoms or molecules can freely move in the simulation domain and switch smoothly from one resolution to another, for instance, based on their spatial coordinates, within the same simulation run. Details for all three techniques have been worked out and reviewed [1]. Additional, highly useful information on extending atomistic simulations via hyperdynamics, parallel replica dynamics, or temperature-accelerated dynamics has been discussed by these authors as well. A sequential scheme where Brownian configuration fields are coupled to macroscopic hydrodynamic governing equations has been used by Liu et al. [2] to study the macroscopic and microscopic characteristics in the fluid flow of dilute polymer solutions in 2D planar channels.

Polymer Networks

Sridhar and Vernerey [3] provided a new framework to describe the nonlinear rheology of transient polymer networks with the so-called chain distribution tensor using recent advances from the transient network theory. This tensor contains quantitative and statistical information of the chain alignment and possible anisotropy that affect network behavior and mechanics. They established shear thickening as a primary result of non-Gaussian chain behavior, addressed a criterion for network fracture, and discussed the role of cross-linker density on viscosity using a "sticky" reptation mechanism. A model for a network of polymer chains inside a composite has been proposed by Chang and Wang [4]. For a CCTO–PDMS composite, the calculation results indicate that the electromechanical properties are greatly affected by internal fillers. With a higher density of CCTO, a higher elastic modulus and dielectric was generally found. Up to a critical level, the interaction between the filler and the matrix contributes to the reinforcement. Dynamical simulations of crosslinked polymer networks in a coarse-grained representation have been performed by Megariotis et al. [5], where entanglements between subchains in the network are represented by slip springs. The ends of the slip springs undergo thermally activated hops between adjacent beads along the chain backbones, which are tracked by kinetic Monte Carlo simulation. In addition, creation/destruction processes are included for the slip springs at dangling subchain ends. Using this approach, the isothermal compressibility of a coarse-grained polymer network as well as its shear stress relaxation modulus are predicted from equilibrium density fluctuations.

Semiflexible Polymers

Recent developments in the theory of individual semiflexible filaments, including crosslinked networks of such filaments, both permanent and transient, have been reviewed by Meng and Terentjev [6]. Local stiffness of polymer chains is instrumental in all structure formation processes of polymers, from crystallization of synthetic polymers to protein folding and DNA compactification. Werlich et al. [7] presented stochastic approximation Monte Carlo simulations, determining the density of states and complete thermodynamic behavior of a model class of single semi-flexible polymer chains, where semiflexibility was introduced by using steric hindrance. Liquid crystalline polymers exhibit a particular richness of behavior that stems from their rigidity and their macromolecular nature. A soft, coarse-grained model has been introduced by Ramirez-Hernandez et al. [8] to explore the interplay of chain stiffness, molecular weight, and orientational coupling, and their roles in the isotropic-nematic transition in homopolymer melts. For this system the orientational coupling rather than excluded volume can be considered as the driving force. The authors, furthermore, studied the structure of polymer mixtures composed of stiff and flexible polymeric molecules. Conditions were found where the systems separate into two phases, one isotropic and the other nematic. Ramirez-Hernandez et al. [8], furthermore, confirm the existence of non-equilibrium states that exhibit sought-after percolating nematic domains, which are of interest for applications in organic photovoltaic and electronic devices.

Polymer Entanglements, Tubes, and Knots

Panagiotou et al. [9] developed topological methods for characterizing the relationship between polymer chain entanglement, knots, and bulk viscoelastic responses. A phenomenon known as "knot-breathing” occurs in biopolymers such as DNA and peptides. Dai and Doyle [10] investigated the effects of intra-chain interactions on knots and found that long-range repulsions can, surprisingly, tighten knots. They tuned the strength of intra-chain repulsion such that a knot was weakly trapped. Driven by thermal fluctuations, the knot switched between tight and loose conformations. They found that coulomb-induced knot trapping could possibly occur in single-stranded DNA and peptides for normal ionic strengths. Within a hybrid model, Moghadam et al. [11] combined a slip-spring model with an "entangled kink dynamics" model for strong uniaxial extensional flows of long entangled polymers in solutions and melts. The model captures the dynamics up to the formation of a kinked or folded state, while the kink dynamics simulation tracks the dynamics from that point forward to complete extension. Unrealistic tension is alleviated by pairing the slip links on one chain with those on a second chain, and allowing some of the large tension on one of the two to be transferred to the second chain, producing non-affine motion of each. Although the entanglement tube framework has achieved remarkable success to describe entangled polymer dynamics, the chain motion assumed in tube theories is still a matter of discussion. Masubuchi [12] compared the performances of two different slip-link simulations. The results imply that bead-spring results are within the scope of the tube framework, whereas the failure of the GLaMM model is possibly due to the assumption of homogeneity along the chain for the fluctuations induced by convective constraint release. The differences between tube-based models used for predicting the linear viscoelasticity of monodisperse linear polymers, in comparison with a large set of experimental data, was the aim of Shchetnikava et al. [13]. The comparison allowed them to highlight and discuss questions related to the relaxation of entangled polymers, such as the importance of the contour-length fluctuations process and how it affects the reptation mechanism, or the contribution of the constraint release process on the motion of the chains. The effect of entanglements on the flow properties of polymeric systems has been addressed in several studies: (i) Properties of the tumbling-snake model for bidisperse entangled polymer melts have been studied both analytically and via Brownian dynamics simulations by Stephanou and Kröger [14], (ii) The thermal lubrication of an entangled polymeric liquid in wall-driven shear flows between parallel plates was investigated using a multiscale hybrid method, coupling molecular dynamics and hydrodynamics by Yasuda [15], (iii) Nonequilibrium molecular dynamics simulations were performed by Sefiddashti et al. [16] to elucidate the origin of stress over- and undershoots.

Polymer Nanocomposites

The fundamental problem of entangled (reptational) homopolymer diffusion in melts and nanocomposite materials in comparison with experiments has been reviewed by Karatrantos et al. [17]. Carbon nanotubes (CNT) with persistence lengths of the order of several microns are one of the most attractive nanomaterials due to their exceptional mechanical, thermal, and electrical properties. They have been widely recognized as one of the strongest materials. Individual CNTs have been adopted to be building blocks of various thermally-stable and viscoelastic materials. CNT buckypaper is one of these assemblies, characterized by the randomly entangled CNT networks. The effects of CNT length on the viscoelasticity and permeability of buckypaper were systematically explored through large-scale coarse-grained molecular dynamics simulations by Shen et al. [18]. Inspired by the hierarchical structure and the outstanding mechanical performance of biological nacre, Liu et al. [19] proposed a multi-layered graphene-–polyethylene nanocomposite as a possible lightweight material with energy-absorbing characteristics. Through coarse-grained molecular dynamics simulations, they studied the mechanical performance of this nanocomposite under spall loading. Results indicate that the maximum contact force during the impact depends on the external surface area of impactors rather than the density of impactors.

Polymers with Special Topology

Theoretical studies on the statistical and dynamical properties of polymers with nontrivial structures in chemical connectivity, and those of polymers with a nontrivial topology, such as knotted ring polymers in solution, have been reviewed by Deguchi and Uehara [20]. Single and double layers of polymer coated surfaces made of ring or linear chains were investigated by means of dissipative particle dynamics (DPD) by Jehser et al. [21].

Finite Element Modeling

Biodegradable stents made of poly-l-lactic acid (PLLA) have a promising prospect thanks to high biocompatibility and a favorable biodegradation period. However, due to the low stiffness of PLLA, polymeric stents have a lower radial stiffness and larger foreshortening. Furthermore, a stent is a tiny meshed tube; hence, it is difficult to make a polymeric stent. Li et al. [22] employed a finite element analysis-based optimization method combined with Kriging surrogate modeling to optimize the stent structure and stent microinjection molding process. Li et al. [23] combined experiments and finite element simulations to study the effect of pre-imposed cyclic loading on the surface instability of silicon rubber under compression. According to these simulations, the experimentally observed multiple creases should be attributed to a thin and stiff layer formed on the surface of silicon rubber after the pre-imposed cyclic loading.

Summary

Multiscale modeling is a heavily active field in modern science with a multidisciplinary character. The actual power of multiscale strategies is only truly appreciated by overcoming traditional barriers between various scientific disciplines. The computational multiscale approaches should eventually fulfill their philosophy, which is to enhance our knowledge of, and ability to control complex processes, even in life sciences. Developing proper multiscale methods is extremely difficult but undeniably represents the future of polymer science as well as computer simulation and modeling. Up to now, multiscale modeling has been under-represented in *Polymers*. On the other hand, there is enormous progress on the front of semiflexible polymers, polymer nanocomposites, and topological interactions that could ultimately become useful in multiscale approaches as well.

## Figures and Tables

**Figure 1:**
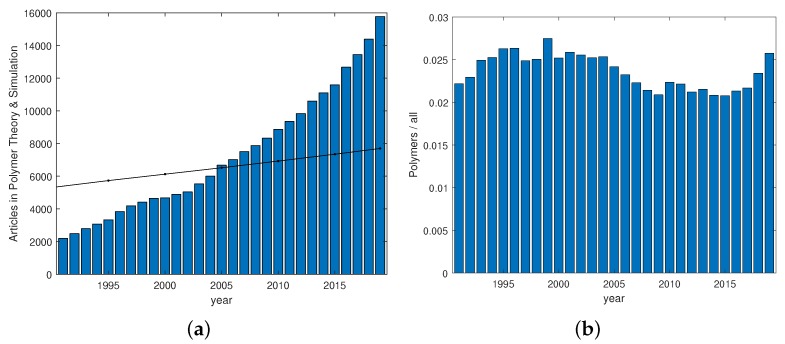
(**a**) Number of peer-reviewed journal articles in the field of polymer theory and simulation within the time range 1991–2019. The black reference line is the population of humans in milliards. (**b**) Fraction of polymer-related articles in the field of theory and simulation. Data from Web of Science (Clarivate Analytics). Approximately 2.5% of all theory and simulation articles are from within the field of polymers.
